# Using Routine Laboratory Markers and Immunological Indicators for Predicting *Pneumocystis jiroveci* Pneumonia in Immunocompromised Patients

**DOI:** 10.3389/fimmu.2021.652383

**Published:** 2021-04-12

**Authors:** Guoxing Tang, Shutao Tong, Xu Yuan, Qun Lin, Ying Luo, Huijuan Song, Wei Liu, Shiji Wu, Liyan Mao, Weiyong Liu, Yaowu Zhu, Ziyong Sun, Feng Wang

**Affiliations:** Department of Laboratory Medicine, Tongji Hospital, Tongji Medical College, Huazhong University of Science and Technology, Wuhan, China

**Keywords:** *Pneumocystis jiroveci* pneumonia, immunosuppressive therapy, T cells, LDH, predictive model

## Abstract

**Background:**

Pneumocystis jiroveci pneumonia (PJP) is the most common opportunistic infection in immunocompromised patients. The accurate prediction of PJP development in patients undergoing immunosuppressive therapy remains challenge.

**Methods:**

Patients undergoing immunosuppressive treatment and with confirmed pneumocystis jiroveci infection were enrolled. Another group of matched patients with immunosuppressant treatment but without signs of infectious diseases were enrolled to control group.

**Results:**

A total of 80 (40 PJP, 40 non-PJP) participants were enrolled from Tongji Hospital. None of the patients were HIV positive. The routine laboratory indicators, such as LYM, MON, RBC, TP, and ALB, were significantly lower in PJP patients than in non-PJP patients. Conversely, LDH in PJP patients was significantly higher than in non-PJP controls. For immunological indicators, the numbers of T, B, and NK cells were all remarkably lower in PJP patients than in non-PJP controls, whereas the functional markers such as HLA-DR, CD45RO and CD28 expressed on CD4^+^ or CD8^+^ T cells had no statistical difference between these two groups. Cluster analysis showing that decrease of host immunity markers including CD3^+^, CD4^+^ and CD8^+^ T cells, and increase of tissue damage marker LDH were the most typical characteristics of PJP patients. A further established model based on combination of CD8^+^ T cells and LDH showed prominent value in distinguishing PJP from non-PJP, with AUC of 0.941 (95% CI, 0.892-0.990).

**Conclusions:**

A model based on combination of routine laboratory and immunological indicators shows prominent value for predicting the development of PJP in HIV-negative patients undergoing immunosuppressive therapy.

## Introduction

Opportunistic infection has become a global pandemic and major public health concern in immunocompromised patients (1, 2). Pneumocystis jiroveci pneumonia (PJP), formerly known as Pneumocystis carinii pneumonia (PCP), is the most common opportunistic infection, causing high mortality and morbidity in developing countries. It is generally viewed that PJP is well known to affect patients infected with HIV, but recently this infection is being increasingly diagnosed in HIV-negative patients, in whom it carries a poorer prognosis (3, 4). Given the serious consequence of PJP, it is important to discover some markers which could be used to predict the occurrence of this disease.

For individuals without HIV infection, immunosuppressive therapies are the main cause of low immunity and the subsequent PJP occurs (5, 6). Previous studies have reported that patients with autoimmune diseases and organ transplantation are the main users of immunosuppressive agents, and these patients are at high risk of PJP due to the status of treatment-related immunosuppression (7–9). Furthermore, absolute peripheral lymphopenia, high doses of corticosteroids with or without combination of other immunosuppressive agents, and concomitant lung disease are strong predictors for the development of PJP, and thus should warrant primary prophylaxis (10). Notably, the CD4^+^ T-cell < 200 cells/μl is a risk factor for PJP in either HIV-infected patients or those with immunosuppressive treatment (7, 11). However, whether other lymphocytes or the function of these lymphocytes could be used in predicting the occurrence of PJP remains obscure. Besides, the cutoff value of these immunological indicators should be further validated.

On the other hand, the laboratory diagnosis of PJP still faces some dilemmas in clinical practice. Given pneumocystis jiroveci cannot be propagated in culture, microscopic visualization of cysts or trophic forms in pulmonary specimens with cytochemical staining (Wright-Giemsa, Gomori methenamine silver (GMS), and Toluidine blue O staining), or immunofluorescent staining with monoclonal antibodies, and/or DNA amplification *via* polymerase chain reaction (PCR) are the standard procedures to detect this pathogen (12–16). Real-time fluorescence quantitative PCR can be used not only to distinguish PJP from other infections but also to determine the relative pathogen load (17, 18). However, when using clinical diagnosis as the standard for diagnosing PJP, the sensitivity of PCR is still insufficient (19). Besides, some pulmonary specimens such as bronchoalveolar lavage fluid are obtained by invasive techniques, carrying an associated risk of complications during collection, especially in patients with respiratory problems (20). Thus, new methods for differential diagnosis of PJP by using non-invasive samples are necessary.

This study aimed to describe the routine laboratory features and immunological characteristics of patients who were undergoing immunosuppressant treatment and developed PJP. We found several indicators, such as lymphocytes, CD4^+^ and CD8^+^ T cells, albumin (ALB) and lactate dehydrogenase (LDH), had predictive value for PJP occurrence. A further established model based on combination of CD8^+^ T cells and LDH produced a prominent effect on predicting the occurrence of PJP.

## Methods

### Study Design and Participants

Between October 2018 and October 2020, the patients who were undergoing immunosuppressant treatment and with suspected pneumocystis jiroveci infection (with symptoms of lung infection and abnormal findings on chest images) were recruited from Tongji Hospital, Tongji Medical College, Huazhong University of Science and Technology. The suspected patients who had positive PCR results of pneumocystis jiroveci and with final PJP diagnosis were enrolled to the study. Another group of patients who were undergoing immunosuppressant treatment (the same treatment dose and duration with PJP group) but without signs of infectious diseases (with no symptom and normal chest image) were enrolled to control group. The clinical information (age, gender, causes of immunodeficiency (autoimmune diseases and organ transplantation undergoing immunosuppressive therapy), underlying condition or illness) and routine laboratory data (WBC, white blood cells; NEU, neutrophils; LYM, lymphocytes; MON, monocytes; EOS, eosinophils; RBC, red blood cells; Hb, hemoglobin; TP, total protein; ALB, albumin; GLB, globulin; A/G, albumin/globulin; LDH, lactate dehydrogenase) were collected from electronic medical records. Laboratory data within one week before the diagnosis of PJP were collected, and no patient received PJP prophylaxis. Patients with missing data and younger than 18 years of age were excluded from the study. This study was approved by the Ethics Committee of Tongji Hospital, Tongji Medical College, Huazhong University of Science and Technology.

### Real-Time Fluorescence Quantitative PCR

Real-time fluorescence quantitative PCR for detecting pneumocystis jiroveci was performed as following (1): pulmonary specimens (sputum and/or bronchoalveolar lavage fluid) were collected and digested with digestive juice (2); nucleic acid was extracted by using Tianlong automatic nucleic acid workstation (3); extracted nucleic acid was added to the prepared reaction system (PANA9600E); and (4) real-time fluorescence quantitative PCR was performed using the following conditions: 95°C for 5 min for denature, 45 cycles of amplification at 95°C for 15 s and 60°C for 45 s, 37°C for 15 s for cooling. The positive pneumocystis jiroveci real-time fluorescence quantitative PCR result was defined if cycle thresholds were < 35.

### Lymphocyte Subset Counting and Phenotype Analysis

Heparinized peripheral blood was collected from study participants. The percentages and absolute numbers of CD4^+^ T, CD8^+^ T, CD19^+^ B and CD3^-^CD56^+^ NK cells were determined by using TruCOUNT tubes and BD Multitest 6-color TBNK Reagent Kit (BD Biosciences) according to the manufacturer’s instructions. In brief, 50 μl of whole blood was labeled with 6-color TBNK antibody cocktail for 15 min in room temperature. After adding 450 μl of FACS Lysing Solution, samples were analyzed with FACSCanto flow cytometer using FACSCanto clinical software (BD Biosciences).

The following monoclonal antibodies were added to 100 μl of peripheral blood: anti-CD45, anti-CD3, anti-CD4, anti-CD8, anti-CD28, anti-HLA-DR, anti-CD45RA, and anti-CD45RO (BD Biosciences). Isotype controls with irrelevant specificities were included as negative controls. All of these cell suspensions were incubated for 20 min at room temperature. After lysing red blood cells, the cells were washed and resuspended in 200 μl of PBS. The percentages of CD28^+^CD4^+^ T cells, CD28^+^CD8^+^ T cells, HLA-DR^+^CD3^+^ T cells, HLA-DR^+^CD8^+^ T cells, CD45RA^+^CD4^+^ T cells, CD45RO^+^CD4^+^ T cells, CD4^+^CD25^+^CD127^-^ Treg cells, CD45RA^+^ Treg cells, and CD45RO^+^ Treg cells were analyzed with FACSCanto flow cytometer.

### Statistical Analysis

Categorical variables were expressed as number (%). Continuous variables were expressed as means ± standards deviation (SD) or median (interquartile range). Comparison was performed using Mann-Whitney *U* test for continuous variables and Chi-square test or Fisher’s exact test for categorical variables. Statistical significance was considered when *P* < 0.05. For the identification of a predictive model, also considering our limited number of patients, we used all indicators with AUC higher than 0.8 for multivariable logistic regression analysis, and the regression equation (predictive model) was obtained. The regression coefficients of the model were regarded as the weights for the respective variables, and a score for each patient was calculated. The performance of predictive models was evaluated by the receiver operating characteristic (ROC) curve analysis. Sensitivity, specificity, positive predictive value (PPV), negative predictive value (NPV), and accuracy, together with their 95% confidence intervals (CI), were calculated. Data were analyzed using SPSS version 25.0 (SPSS, Inc., Chicago, IL, USA) and GraphPad Prism version 8 (GraphPad Software, San Diego, CA, USA).

## Results

### The Clinical and Demographic Characteristics of the Participants

A total of 40 PJP patients (26 males, 14 females; medium age, 52 years; IQR, 23-73 years) were included in the study. Another 40 patients who received immunosuppressive treatment but without signs of infectious diseases, with matched gender, age, and underlying conditions as PJP patients, were enrolled as control group (non-PJP). The main clinical and demographic characteristics of the patients were summarized in **Table 1**. Autoimmune disease patients who were undergoing immunosuppressive treatment were most common source of PJP, followed by patients with organ transplantation. Many underlying conditions, such as hypertension, smoking, and chronic kidney disease, were commonly noted in PJP patients. None of the PJP patients had positive HIV status.

**Table 1:** The clinical and demographic characteristics of study participants.

	PJP patients (n = 40)	non-PJP patients (n = 40)
Age, years, median (25th - 75th centiles)	52 (44-61)	48 (43-56)
Males, n (%)	26 (65)	26 (65)
**Patient sources, n (%)**		
Solid organ transplant	7 (17.5)	6 (15)
Autoimmune disease	33 (82.5)	34 (85)
**Clinical presentation**		
Cough	22 (55)	0 (0)
Fever	12 (30)	0 (0)
Chest distress	11 (27.5)	0 (0)
**Radiological findings**		
Lung shadow	32 (80)	0 (0)
Lung nodules	7 (17.5)	0 (0)
Pleural effusion	18 (45)	0 (0)
**Maintenance immunosuppressive regimen, n (%)**		
Corticosteroids (convert to methylprednisolone) ≥ 20mg/day	39 (97.5)	38 (95)
Tacrolimus	6 (15)	4 (10)
Cyclophosphamide	6 (15)	9 (22.5)
Mycophenolate Mofetil	1 (2.5)	3 (7.5)
**Underlying condition or illness, n (%)**		
Smoking	9 (22.5)	8 (20)
Drinking	3 (7.5)	3 (7.5)
HIV	0 (0)	0 (0)
Diabetes mellitus	5 (12.5)	6 (15)
Hypertension	17 (42.5)	16 (40)
Chronic pulmonary disease	4 (10)	0 (0)
Chronic kidney disease	13 (32.5)	40 (100)
Chronic heart failure	1 (2.5)	1 (2.5)

PJP, Pneumocystis jiroveci pneumonia; HIV, human immunodeficiency virus; Data are presented as number (percentage), means ± SD, or medians (25th - 75th centiles).

### Routine Laboratory Findings and Immunological Characteristics of PJP Patients

We observed that PJP patients and non-PJP controls showed no statistical difference in both WBC and NEU count. However, the levels of LYM, MON, RBC and Hb were significantly lower in PJP group than in non-PJP control group (**Table 2**). Moreover, many biochemical indicators, including TP, ALB, and A/G, were also significantly lower in PJP patients than in non-PJP controls. Conversely, the level of LDH in PJP patients was significantly higher than in non-PJP controls (**Table 2**).

**Table 2:** Routine laboratory findings and immunological results of enrolled patients.

	PJP patients	non-PJP patients	*P-Value	AUC
Routine blood examination				
WBC (×10^9^/L)	8.23 (3.99-12.22)	8.15 (5.39-10.91)	0.683	
NEU (×10^9^/L)	7.13 (3.28-10.41)	6.52 (4.07-8.96)	0.613	
LYM (×10^9^/L)	0.53 (0.13-2.12)	0.97 (0.45-2.90)	<0.001	0.812
MON (×10^9^/L)	0.33 (0.06-1.07)	0.45 (0.21-0.70)	0.043	0.632
EOS (×10^9^/L)	0.00 (0.00-0.13)	0.01 (0.00-0.22)	0.183	
RBC (×10^12^/L)	3.33 (2.48-5.80)	3.96 (3.22-4.70)	0.001	0.713
Hb (g/L)	101.2 (76.3-177.4)	116.6 (89.2-144.1)	0.004	0.687
**Routine biochemical examination**				
TP (g/L)	56.9 (47.0-103.9)	62.8 (54.2-71.3)	0.006	0.679
ALB (g/L)	28.7 (21.6-50.3)	36.9 (31.5-42.4)	<0.001	0.819
GLB (g/L)	28.3 (20.9-49.2)	26.1 (21.3-30.8)	0.108	
A/G (g/L)	0.99 (0.58-2.22)	1.44 (1.17-1.70)	<0.001	0.766
LDH (U/L)	473 (219-692)	229 (151-369)	<0.001	0.823
**Lymphocyte subsets**				
CD3^+^ T cells (%)	64.58 (43.17-88.74)	75.32 (64.57-86.08)	<0.001	0.736
CD3^+^ T cell number (/μl)	261 (89-1187)	863 (398-2209)	<0.001	0.911
CD4^+^ T cells (%)	30.96 (18.53-49.49)	39.02 (18.12-49.92)	0.013	0.662
CD4^+^ T cell number (/μl)	117 (14-582)	397 (225-1817)	<0.001	0.902
CD8^+^ T cells (%)	30.45 (15.99-46.44)	33.65 (21.31-45.99)	0.184	
CD8^+^ T cell number (/μl)	118 (23-469)	395 (150-1050)	<0.001	0.888
CD19^+^ B cells (%)	18.47 (6.55-25.01)	11.92 (4.12-19.72)	0.016	0.656
CD19^+^ B cell number (/μl)	64 (0-332)	101 (1-546)	0.016	0.656
CD56^+^ NK cells (%)	12.84 (2.82-37.03)	9.10 (2.23-37.79)	0.039	0.634
CD56^+^ NK cell number (/μl)	58 (5-296)	98 (23-729)	0.006	0.679
CD4^+^ T cell number (/μl)/CD8^+^ T cell number (/μl)	1.39 (0.39-1.78)	1.13 (0.47-3.39)	0.718	

PJP, Pneumocystis jiroveci pneumonia; AUC, area under the curve; WBC, white blood cells; NEU, Neutrophils; LYM, lymphocytes, MON, monocytes; EOS, Eosinophils; RBC, red blood cells; Hb, hemoglobin; TP, total protein; ALB, albumin; GLB, globulin; A/G, albumin/globulin; LDH, lactate dehydrogenase; *Comparisons were performed between PJP group and non-PJP group using Mann-Whitney U test chi-square test. Data are presented as number (percentage), means ± SD, or medians (2.5th - 97.5th centiles).

Given the percentages of CD3^+^, CD4^+^, and CD8^+^ T cells were significantly lower in PJP patients than in non-PJP controls, the percentages of CD19^+^ B and CD3^-^CD56^+^ NK cells were relatively significantly higher in PJP patients in comparison to non-PJP controls. However, the numbers of CD3^+^ T, CD4^+^ T, CD8^+^ T, CD19^+^ B, and CD3^-^CD56^+^ NK cells were all remarkably lower in PJP patients than in non-PJP controls. There was no statistical difference in the ratio of CD4^+^ T cells to CD8^+^ T cells between the two groups, supporting the evidence that the loss of CD4^+^ and CD8^+^ T cells was parallel in PJP patients (**Table 2**).

**Figure 1:**
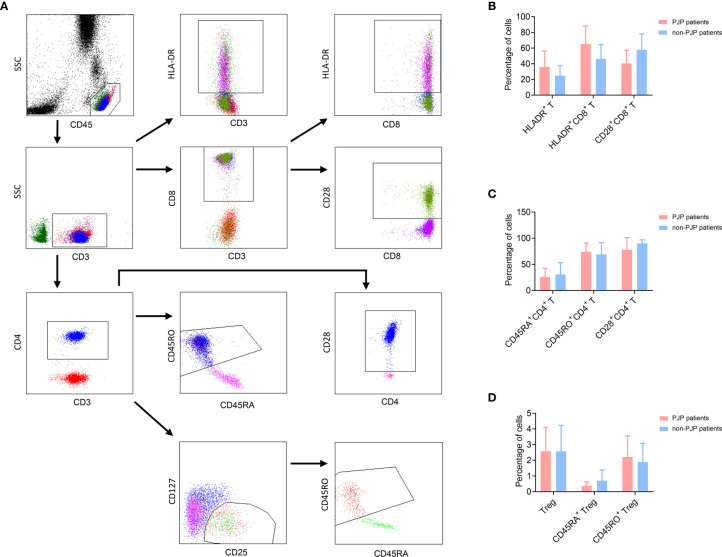
Phenotype analysis of T cell subsets. **(A)** The gating strategies of phenotype analysis of T cell subsets. **(B)** The percentages of CD28^+^CD8^+^ T cells, HLA-DR^+^CD3^+^ T cells, HLA-DR+CD8+ T cells in PJP and non-PJP patients are shown in histogram. Data are expressed as mean ± SD. **(C)** The percentages of CD28^+^CD4^+^ T cells, CD45RA^+^CD4^+^ T cells, CD45RO^+^CD4^+^ T cells in PJP and non-PJP patients are shown in histogram. Data are expressed as mean ± SD. **(D)** The percentages of CD4^+^CD25^+^CD127^-^ Treg cells, CD45RA^+^ Treg cells and CD45RO^+^ Treg cells in PJP and non-PJP patients are shown in histogram. Data are expressed as mean ± SD.

The functional markers of lymphocytes were further determined. We observed that the activation (HLA-DR) and memory (CD45RO) markers expressed on CD4^+^ or CD8^+^ T cells were higher in PJP group, while the expression of CD28 on both CD4^+^ and CD8^+^ T cells was lower in PJP group, compared with non-PJP control group. However, these differences failed to achieve statistical significance. There was no significant difference in the percentage of either Treg or CD45RA^+^ Treg between PJP and non-PJP patients (**Figure 1**, **Table 3**).

**Table 3:** Analysis of the phenotype of lymphocytes in PJP and non-PJP patients.

	PJP patients (n = 40)	non-PJP patients (n = 40)	*P-Value
Age (years)	52 (23-73)	49 (39-60)	0.383
Males, n%	26 (65)	26 (65)	0.258
**Lymphocyte subsets**			
CD28^+^ CD4^+^ T cells (%)	83.74 (32.91-99.50)	90.18 (83.27-97.08)	0.243
CD28^+^CD8^+^ T cells (%)	40.42 (23.6157.22)	57.99 (37.74-78.24)	0.079
HLA-DR^+^CD3^+^ T cells (%)	32.14 (16.46-71.84)	24.64 (11.69-27.79)	0.400
HLA-DR^+^CD8^+^ T cells (%)	65.02 (41.85-88.19)	46.22 (27.79-64.65)	0.079
CD45RA^+^CD4^+^ T cells (%)	25.86 (9.22-42.50)	30.80 (8.44-53.17)	0.841
CD45RO^+^CD4^+^ T cells (%)	74.14 (57.50-90.78)	69.20 (46.86-91.55)	0.842
CD4^+^CD25^+^CD127^-^ cells (%)	2.58 (1.06-4.09)	2.75 (1.11-4.40)	0.905
CD45RA^+^ Treg cells (%)	0.37 (0.10-0.63)	0.33 (0.11-1.93)	0.497
CD45RO^+^ Treg cells (%)	2.21 (0.87-3.55)	2.02 (0.84-3.21)	0.661

PJP, Pneumocystis jiroveci pneumonia; *Comparisons were performed between PJP group and non-PJP group using Mann-Whitney U test chi-square test. Data are presented as number (percentage), means ± SD, or medians (2.5th - 97.5th centiles).

**Figure 2:**
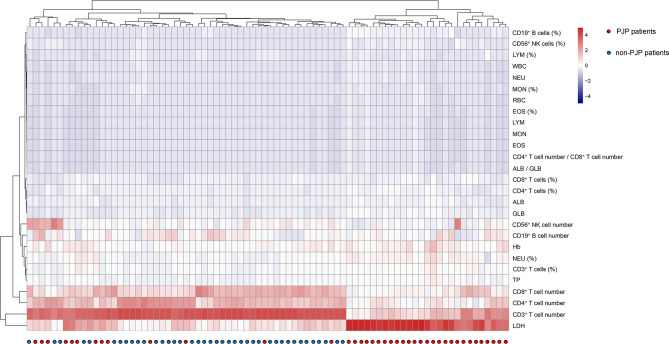
Clustering analysis of routine laboratory and immunological indicators in PJP and non-PJP patients. On the y axis are indicator values after z-scoring, and on the x axis are individual patients. Red-white-blue squares represent z-scoring values. WBC, white blood cells; NEU, neutrophils; LYM, lymphocytes; MON, monocytes; EOS, eosinophils; RBC, red blood cells; Hb, hemoglobin; TP, total protein; ALB, albumin; GLB, globulin; LDH, lactate dehydrogenase.

The overall profile of routine laboratory results and immunological indicators in enrolled patients was shown in heatmap (**Figure 2**). Hierarchical cluster analysis found that these indicators showed potential in distinguishing these two conditions. In comparison to non-PJP patients, PJP patients displayed typical laboratory pattern, characterizing as the decrease of host immunity markers including CD3^+^, CD4^+^ and CD8^+^ T cell number, and the increase of tissue damage marker LDH.

### Development of the Predictive Model for Discriminating Between PJP and Non-PJP Patients

**Figure 3:**
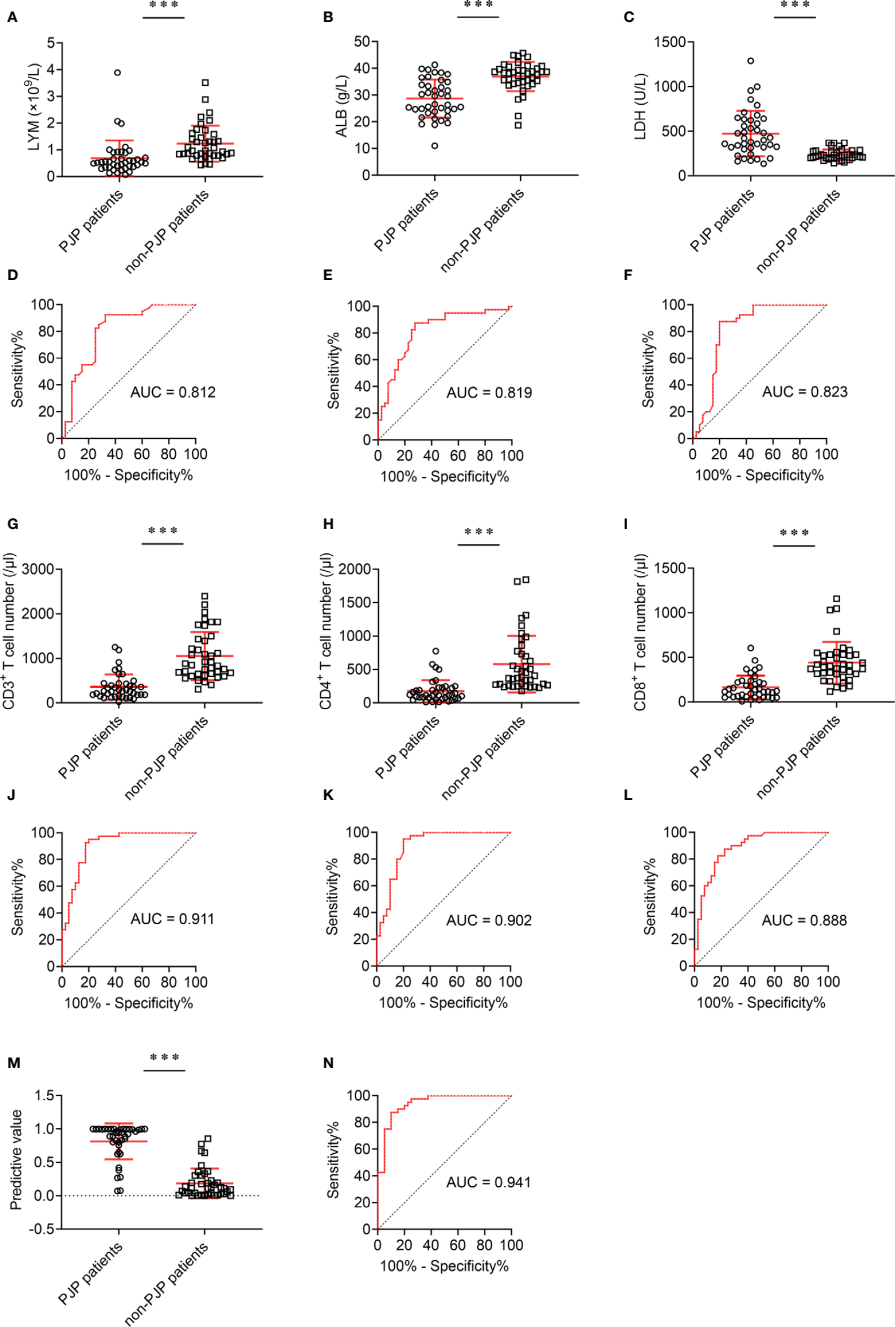
The effect of different indicators on discriminating between PJP and non-PJP. **(A)** Scatter plots showing the values of LYM, **(B)** ALB, and **(C)** LDH in PJP and non-PJP patients. **(D)** ROC analysis showing the performance of LYM, **(E)** ALB, and **(F)** LDH in distinguishing PJP and non-PJP patients; **(G)** Scatter plots showing the values of CD3^+^ T cells, **(H)** CD4^+^ T cells, and **(I)** CD8^+^ T cells in PJP and non-PJP patients. **(J)** ROC analysis showing the performance of CD3^+^ T cells, **(K)** CD4^+^ T cells, and **(L)** CD8^+^ T cells in distinguishing PJP from non-PJP patients. **(M)** Scatter plots showing the value of the diagnostic model in PJP and non-PJP patients. **(N)** ROC analysis showing the performance of the diagnostic model in distinguishing PJP and non-PJP patients. Horizontal lines indicate the mean ± SD of each group. ***p < 0.001 (Mann-Whitney *U* test). LYM, lymphocytes; ALB, albumin; LDH, lactate dehydrogenase; AUC, area under the curve.

The effect of these indicators with statistical significance on discriminating between PJP and non-PJP was further analyzed. Four indicators, including lymphocyte count, ALB, LDH and CD8^+^ T cell number, had potential value in distinguishing PJP and non-PJP, with AUC between 0.8 and 0.9. Notably, two indicators, including CD3^+^ T cell number and CD4^+^ T cell number, performed better in distinguishing these two conditions, with AUC higher than 0.9 (**Figure 3**).

To develop the predictive models based on the combination of various indicators for distinguishing PJP patients from non-PJP controls, also considering the limited number of patients, we selected all indicators with AUC higher than 0.8 for further univariate and multivariate analyses. On multivariable logistic regression analysis, LDH and CD8^+^ T cell number were chosen as predictive model markers (**Table 4**). Based on regression coefficients, we established a predictive model in distinguishing PJP patients from non-PJP controls as follow: P = 1/[1 + e^-(0.012*LDH - 0.011* CD8+ T cell number - 0.403)^]. P, predictive value; e, natural logarithm. The score of each patient was calculated, and a higher score would predict greater likelihood of PJP.

**Table 4:** Univariate and multivariate analyses of risk factors associated with infection of PJP.

	Univariate analysis (n = 80)			Multivariate analysis (n = 80)		
	OR	95% CI	*P*-Value	OR	95% CI	*P*-Value
LYM (×10^9^/L)	0.206	0.074-0.573	<0.001			
ALB (g/L)	0.816	0.743-0.896	<0.001			
LDH (U/L)	1.013	1.007-1.020	<0.001	1.012	1.004-1.019	0.002
CD3^+^ T cell number (/μl)	0.995	0.992-0.997	<0.001			
CD4^+^ T cell number (/μl)	0.992	0.988-0.996	<0.001			
CD8^+^ T cell number (/μl)	0.989	0.985-0.994	<0.001	0.989	0.983-0.995	<0.001

PJP, Pneumocystis jiroveci pneumonia; OR, odds ratio; CI, conﬁdence interval; LYM, lymphocytes; ALB, albumin; LDH, lactate dehydrogenase.

### Comparing the Performance of 2-Indicator Model and Single Indicator

The predictive model based on combination of LDH and CD8^+^ T cell number performed best in distinguishing PJP from non-PJP, with AUC of 0.941 (95% CI, 0.892-0.990) (**Figure 3**). When 0.373 was used as the cutoff value, the sensitivity and specificity of 2-indicator model were 90.00% and 87.50% respectively, with a predictive accuracy of 88.75% (**Table 5**). CD3^+^ T cell number presented an AUC of 0.911 (95% CI, 0.847-0.946), with a sensitivity of 92.5% and a specificity of 82.5% when 497 was used as the cutoff value. CD4^+^ T cell number presented an AUC of 0.902 (95% CI, 0.832-0.972), with a sensitivity of 95.00% and a specificity of 80.00% when 230 was used as the cutoff value. CD8^+^ T cell number presented an AUC of 0.888 (95% CI, 0.817-0.960), with a sensitivity of 82.5% and a specificity of 82.5% when 241.5 was used as the cutoff value (**Table 5**).

**Table 5:** Performance of indicators and model in predicting infection of PJP.

Variable	Value (95% CI)						
	LYM (×10^9^/L)	ALB (g/L)	LDH (U/L)	CD3^+^ T cell number (/μl)	CD4^+^ T cell number (/μl)	CD8^+^ T cell number (/μl)	2-Maker Model
AUC	0.812 (0.715 - 0.910)	0.819 (0.724 - 0.914)	0.823 (0.722 - 0.925)	0.911 (0.847 - 0.976)	0.902 (0.832 - 0.972)	0.888 (0.817 - 0.960)	0.941 (0.892 - 0.990)
Cut-off Value	0.645	33.35	296.5	497	230	241.5	0.373
Sensitivity (%)	92.50	87.50	80.00	92.50	95.00	82.50	90.00
Specificity (%)	67.50	72.50	87.50	82.50	80.00	82.50	87.50
PPV (%)	74.00	76.09	86.49	84.09	82.61	82.50	87.80
NPV (%)	90.00	85.29	81.40	91.67	94.12	82.50	89.74
Accuracy (%)	80.00	80.00	83.75	87.50	87.50	82.50	88.75

PJP, Pneumocystis jiroveci pneumonia; CI, conﬁdence interval; LYM, lymphocytes; ALB, albumin; LDH, lactate dehydrogenase; AUC, area under the curve; PPV, positive predictive value; NPV, negative predictive value.

## Discussion

PJP is a common opportunistic pathogen which causes severe infections and high mortality in immunocompromised patients (21, 22). It is noteworthy that, approximately 50% of adults may carry pneumocystis jiroveci, whereas only individuals with low immunity will develop into active disease, further supporting the evidence that PJP can be used as a symbol of immunosuppression (22). Recently, more and more PJP patients are reported in HIV-negative patients, with the increase of using immunosuppressants in clinical practice (23, 24). However, which laboratory indicators can be used to predict the development of PJP in patients during immunosuppressive treatment remains obscure, and the answer to this is obviously critical to timely prophylaxis and improving mortality (25). In this study, after matching age, gender, immunosuppressant exposure, and underlying conditions or illnesses, we compared the characteristics of routine laboratory tests and immunological indicators of patients with PJP to those of patients with non-PJP. The 2-indicator model had a prominent value for predicting the occurrence of PJP in patients undergoing immunosuppressive treatment.

It has been reported that many conditions, such as old age, underlying diseases, HIV infection, use of multiple immunosuppressants, have been identified as risk factors for PJP (26, 27). However, most of these conditions are unfeasible to quantify in clinical practice. In the present study, we found that CD4^+^ T cell number was an important marker in the prediction of PJP, and this is in accordance with previous findings (24). Nevertheless, the cutoff value of CD4^+^ T cell number in our study was slightly different from previous reports, which may be caused by the heterogeneity of the patients (28, 29). Surprisingly, we found that CD3^+^ and CD8^+^ T cell numbers also had good performance in predicting the development of PJP, which was rarely reported before. The AUC of CD3^+^ T cell number was even higher than CD4^+^ T cell number. Previous studies focused on investigating the characteristics of PJP in patients with HIV infection, causing that the decline of CD4^+^ T cell number was the main manifestation of the disease (30, 31). Thus, CD4^+^ T cell number was recognized as most important laboratory indicator for predicting the development of PJP (24, 28). Differently, this study aimed to investigate the characteristics of PJP in HIV-negative patients. We found despite CD4^+^ T cells, the lymphocytes including CD3^+^ T cells, CD8^+^ T cells, B cells and NK cells were all decreased in PJP patients due to the use of immunosuppressive agents. These data suggest that the characteristics of immunological indicators are different in patients with different causes of immunodeficiency. Consistent with this notion, another study focused on the laboratory tests in PJP patients with organ transplantation observed similar data (16), supporting the idea that CD3^+^ and CD8^+^ T cell numbers are prominent indicators for predicting PJP development. This study confirms the idea that CD3^+^ T cell number is an important marker for reflecting immune status and need be monitored in patients undergoing immunosuppressive therapy (32). Moreover, this theory may be expanded to other fields, such as for predicting the occurrence of other opportunistic infections in cancer patients undergoing chemotherapy.

Previous studies have shown that over 90% of PJP patients exceeded the reference range of biochemical indicators such as CRP, ESR, LDH, and β-glucan (33–35). In accordance with these findings, we observed that an increase of serum LDH was commonly noted in PJP patients, which was probably due to lung injury. However, the performance of using LDH for prediction PJP occurrence was limited, which was consistent with previous study showing that LDH level had a high sensitivity for PJP but a limited specificity. After all, LDH was commonly elevated in many diseases such as heart and hepatobiliary disorders (36, 37). Conversely, the level of ALB was decreased in PJP patients, which suggested that ALB had some potential in predicting the development of PJP. It is because that the decrease of ALB is one of the signs of low immunity and commonly noted in patients with opportunistic infections (38, 39). The inflammatory indicators, such as WBC, neutrophil and C-reactive protein, would have very limited value in the prediction of PJP, as these indicators are non-specific and increased in other lung infections besides PJP (34).

Although some studies have focused on the risk factors associated with PJP, modeling the interrelationship among factors is rare (40, 41). To our best knowledge, this is the first work to establish a mathematical model for predicting PJP occurrence in HIV-negative patients who are undergoing immunosuppressive therapy. To our surprise, CD8^+^ T cells, but not CD3^+^ or CD4^+^ T cells, were incorporated into the predictive model, which suggested that CD8^+^ T cells and LDH have synergic effect on predicting PJP occurrence.

Several limitations of the study should be mentioned. First, it has to note that the major limitation of this study is the small number of patients, due to the rarity of the disease. A further prospective study should be carried out to verify the performance of the prediction model. Second, the model we established in this study can only be used to predict PJP infection in patients undergoing immunosuppressive therapy, but cannot be used for distinguishing PJP infection from other opportunistic infections. Third, this model cannot be used for predicting PJP infection in HIV-positive patients. Fourth, given that this was a retrospective study and some laboratory results were obtained in the course of PJP, the laboratory data could be affected by the illness. Thus, the accuracy of this predictive model may have bias in real clinical practice.

Collectively, this study has addressed the characteristics of routine laboratory tests and immunological indicators in PJP patients. Our data suggest that many laboratory indicators, such as CD3^+^, CD4^+^, CD8^+^ T cell numbers and LDH, can serve as risk factors for PJP occurrence, and a model based on combination of CD8^+^ T cell number and LDH shows prominent value for predicting the development of PJP in HIV-negative patients undergoing immunosuppressive therapy.

## Data Availability Statement

The raw data supporting the conclusions of this article will be made available by the authors, without undue reservation.

## Ethics Statement 

The studies involving human participants were reviewed and approved by Ethics Committee of Tongji Hospital, Tongji Medical College, Huazhong University of Science and Technology. Written informed consent for participation was not required for this study in accordance with the national legislation and the institutional requirements.

## Author Contributions

GT, ZS and FW conceived of the research, designed the study, interpreted data, and wrote the manuscript. YL, ST, XY, QL, GT, LM, and HS contributed to the acquisition of clinical data. GT, YZ, SW, WYL and WL recruited the participants, performed experiments, and analyzed data. All authors contributed to the article and approved the submitted version.

## Funding

This work was supported in part by grants from National Natural Science Foundation (81401639), and in part by National Mega Project on Major Infectious Disease Prevention of China (2017ZX10103005-007).

## Conflict of Interest

The authors declare that the research was conducted in the absence of any commercial or financial relationships that could be construed as a potential conflict of interest.
